# Population-level communication interventions for breastfeeding: Protocol for a scoping review

**DOI:** 10.12688/hrbopenres.14088.2

**Published:** 2025-11-17

**Authors:** Jennifer Hanratty, Karl McGrath, Karen Matvienko-Sikar, Elaine Lehane, Aoife Long, Caroline Rawdon, Patricia Leahy-Warren

**Affiliations:** 1 Centre for Effective Services, Dublin, Leinster, Ireland; 2 School of Public Health, University College Cork, County Cork, Ireland; 3 School of Nursing and Midwifery, University College Cork, County Cork, Ireland

**Keywords:** breastfeeding, social change communication, behaviour change, mass media, scoping review

## Abstract

**Objective:**

The objective for this scoping review is to systematically identify and map the breadth, type, and characteristics of population-level communication interventions on breastfeeding.

**Introduction:**

Breastfeeding is the human biological norm and is well established to be foundational for health throughout the life course. Population-level communication interventions, or mass communication interventions can support positive social and cultural norms and practices that enable breastfeeding. To inform the development of effective mass communication interventions there is a need to systematically identify, analyse and synthesise the existing evidence to inform such guidance.

**Inclusion criteria:**

We will include evidence on the development, implementation and effectiveness of population-level communications interventions in high development index countries (including traditional mass media, social media or other mass communication interventions) aimed at influencing breastfeeding at scale. We will include empirical evidence from primary studies and evidence synthesis.

**Methods:**

We will search databases for primary studies and reviews, written in English language, from 1990 onwards: PubMed, Embase, EBSCO, PsycInfo, MIDIRS and ProQuest. We will conduct forward and reverse citation searching of included studies and reviews and grey literature searches via web searches, OSF, and contact with known authors/ organisations in the field. The search will be undertaken in January/February 2025.

Screening and data extraction will be conducted using EPPI reviewer web (ER6), making use of automation tools to support efficiency. Disagreements will be discussed until a consensus is reached or a third author makes a final decision.

Data extraction will include information on study location, target and subject populations, message content, message framing, declared conflicts of interest and underlying theory used.

## Introduction

The importance of breastfeeding for children, women and society is well established, bringing with it a myriad of health, developmental ( Victora *et al*., 2016), environmental ( Bai & Alsaidi, 2024) and economic benefits ( Hansen, 2016). The World Health Organisation (WHO) recommends that children are exclusively breastfed for the first 6 months of life, and continue to be breastfed after complementary foods are introduced to at least 2 years of age or beyond ( WHO, 2025). Yet, breastfeeding rates fall well below both biologically feasible rates and the WHO global infant feeding recommendations in most countries. Past estimates suggested only 37% of infants younger than 6 months were exclusively breastfed in low- and middle-income countries (LMIC), with even lower rates in high income countries (HIC) ( Hansen, 2016; Victora *et al*., 2016). Global trends in breastfeeding rates over time indicate some progress in HIC in the past 20 years, but rates still fall far below in LMIC ( Neves *et al*., 2021). In Ireland, the situation is especially concerning. A recent report by the World Breastfeeding Trends Initiative (WTBi) described Ireland’s breastfeeding rates as one of the lowest in the world. So much so that in 2021, exclusive breastfeeding rates were as low as 37% by hospital discharge, roughly 3 days after the mother had given birth with no current national data available on breastfeeding rates beyond this point ( WBTi, 2023).

Social and cultural norms, beliefs and practices strongly influence breastfeeding rates ( The Lancet, 2016). Pérez-Escamilla *et al.*’s (2023) conceptual model of the components of an enabling environment illustrate that breastfeeding decisions and practices are influenced over time by many interacting factors at multiple levels ( Pérez-Escamilla *et al*., 2023; Rollins *et al*., 2016). At a structural level, the context for breastfeeding is shaped by cultural attitudes, social norms and market forces, amongst other factors ( Pérez-Escamilla *et al*., 2023; Rollins *et al*., 2016). For example, in Ireland, the WTBi reported that formula feeding is the norm for infants rather than breastfeeding. Moreover, Ireland is a major producer of infant commercial milk formula and the economic benefits of mass producing infant commercial milk formula potentially creates a conflict of interest at a political level ( WBTi, 2023). Given the many and multi-level factors that influence breastfeeding decisions and practices, breastfeeding should be seen as a collective societal responsibility rather than the sole responsibility of mothers, thus requiring multi-level, multi-component interventions ( Pérez-Escamilla *et al*., 2023; Rollins *et al*., 2016; WBTi, 2023) to sustain and accelerate improvements in breastfeeding rates in high income countries.

Population-level communication interventions can form part of a multi-level, multi-component approach to improving breastfeeding rates. Such interventions can support positive social and cultural norms and practices that enable breastfeeding. Communication through provision of information about importance of health behaviours, their antecedents and their outcomes, and how to perform health behaviours are typically the most commonly used behaviour change techniques used in complex behaviour change interventions, including in those aiming to support optimal infant feeding Eidhin *et al*. (2025); ( Matvienko-Sikar *et al*., 2019). Providing information by itself is rarely sufficient to change behaviour. Efforts to ‘promote’ breastfeeding by providing information about its importance, without addressing the barriers to breastfeeding has resulted in something of a backlash against a perceived pressure to breastfeed ( Entwistle, 2018; UNICEF, 2017). Given the challenges of communication about breastfeeding, evidence-based guidance on how to communicate effectively would be welcome. Preliminary searches conducted in preparation for this scoping review protocol suggest there is currently little available guidance on population-level communication interventions for breastfeeding specifically.

Mass media campaigns are an example of a population-level communication intervention. Mass media campaigns may be a component of a larger package of interventions that could improve breastfeeding outcomes and population health ( Pérez-Escamilla *et al*., 2023; Rollins *et al*., 2016; WBTi, 2023). For the purpose of this review, we define mass media campaigns as population-level communication interventions that actively and systematically use mass media for mass communications with the intent of influencing the health behaviour of large target audience(s) or population(s) (for a full explanation of this definition and its key terms, see Box 1). Mass media campaigns have long been recognised as important tools in public health promotion ( Flay *et al*., 1980; Kreps & Maibach, 2008; Tones *et al*., 1990), that can target single or multiple levels of health determinants, that can be used alone or in combination with other interventions, and which can influence the health behaviours of populations ( Abroms & Maibach, 2008; Maibach *et al*., 2007; Wakefield *et al*., 2010).

**Box 1. Key concepts, terms and approaches relevant to this scoping review**

We outline our definitions of the core terms used in this review below.

     
**Population-level communication interventions** refer to interventions that seeks to communicate with the population at scale to influence health behaviours ( Stead *et al*., 2019). Mass communication campaigns are a type of population-level communication interventions.

     
**Mass communication** refers to the transmission of messages through one or more media to large audiences or populations. **Media** are the technologies that provide the means of transmitting such messages ( Deuze, 2020).

     
**Mass media** refers to technologies that can transmit messages almost instantaneously to large audiences across great geographic distances. Common mass media technologies include television, newspapers, film and radio ( Storey *et al*., 2011).

     
**Social media** are a form of mass media. Social media does not have a definitive definition ( Morse & Brown, 2022; Orchard & Nicholls, 2022), though they typically have the ability to transmit messages across geographical distances to potential large audiences. Social media tend to have greater capacity for users to interact, generate, share their own content, and to target specific audiences more precisely that mass media ( Storey *et al*., 2011).

     
**Mass media campaigns** actively and systematically use mass media to communicate at scale with the intent of influencing the health behaviour of large target audience(s) or population(s).

In this review, an eligible intervention would be, for example, a mixed media campaign with tv ads, radio spots and social media content with specific messaging targeted to specific audiences.

Ineligible interventions would be those that require the target audience to seek out or opt in to receive campaign information. For example, a website that passively provides information, without an accompanying ‘push’ to advertise or otherwise engage people with the information or an mHealth intervention requiring the audience to opt-in to receive information, would be ineligible for this scoping review.

From a socioecological perspective, mass media campaigns can influence health behaviours through three main fields of influence: (1) influencing people, (2) influencing places, and (3) influencing the interaction between people and places ( Abroms & Maibach, 2008). By targeting people, mass media campaigns can directly or indirectly influence health behaviours by changing individual-level attributes (like knowledge, skills, beliefs), social network level outcomes (like social support from family and peers), or population or community-level outcomes (like social norms or cultural attitudes and practices) ( Abroms & Maibach, 2008; Maibach *et al*., 2007). By targeting places, mass media campaigns attempt to indirectly influence health behaviours by advocating for changes in physical and social structures (like workplace spaces and policies), the availability of products and services to support a health behaviour, or the cultural and media messages in an environment (like those transmitted in advertisements). These place-based factors can be targeted at local or distal levels ( Abroms & Maibach, 2008; Maibach *et al*., 2007). From this socio-ecological perspective, communications campaigns have typically sought to directly influence individual-level factors, rather than influencing indirectly by targeting change in the wider environments in which individuals live and work ( Abroms & Maibach, 2008; Maibach *et al*., 2007).

This review represents the first step towards developing guidance to inform the development of population-level communications interventions for breastfeeding in HICs by scoping the relevant evidence from very highly developed countries. The review will place a particular focus on the development of campaigns, their content, target audiences and theoretical underpinnings.

### Existing evidence syntheses

A preliminary search of PUBMED, OSF, the Cochrane Database of Systematic Reviews, The Campbell Library and *JBI Evidence Synthesis and google scholar* was conducted. We identified a number of existing evidence syntheses related to the topic of our scoping review. While there is overlap with our proposed scoping review, none of the reviews identified examine the evidence for the purpose of informing guidance on the design and development of mass media campaigns ( Barnett *et al*., 2022; Darajat *et al*., 2022; Eppes *et al*., 2023; Galvão *et al*., 2021; Gavine *et al*., 2022; Graziose *et al*., 2018; Henderson & Gow, 2023; Lamstein *et al*., 2014; Mahumud *et al*., 2022; Morse & Brown, 2022; Orchard & Nicholls, 2022; Schmidt, 2013; Susilawati & Wijhati, 2021; Tang *et al*., 2019; Underwood, 2021; Wakefield *et al*., 2010). For example, the Wakefield *et al*. (2010) systematic review examined the effectiveness of mass media campaigns in the context a wide range of health behaviours. This systematic review is a useful primer on the mechanisms of change and the factors that influence success in a broad sense, however, it does not focus specifically on breastfeeding behaviour. Orchard and Nicholls (2022) systematic review examined the impact of social media on breastfeeding but focused primarily on online communities and supports; it did not examine the use of social media as a channel for delivering a planned campaign. Darajat *et al.*’s (2022) systematic review of social behaviour change communications to preventing stunting included breastfeeding communications campaigns but this was not the primary focus of their analysis. Similarly, Mahumud *et al.*’s (2022) systematic review of behaviour change communications interventions for improving young child nutrition does include studies on mass media campaigns but breastfeeding is not the primary focus.

One systematic review that does focus specifically on the development, implementation and effectiveness of mass media campaigns for infant feeding outcomes is by Graziose *et al*. (2018). Their review was limited to research from low- and middle-income countries. Our proposed scoping review will complement this existing review with a focus on evidence produced in the context of countries ranked as ‘very high’ on the human development index.

No other published current or underway systematic reviews or scoping reviews on the topic were identified.

## Rationale and objectives

The design and implementation of health communications can significantly affect their influence ( Abroms & Maibach, 2008; Keller & Lehmann, 2008; Maibach *et al*., 2023; Wakefield *et al*., 2010). While the findings across existing systematic reviews highlight that mass media interventions for breastfeeding exist, and can produce positive outcomes, including improved exclusive breastfeeding rates and improved child nutritional outcomes ( Mahumud *et al*., 2022; Orchard & Nicholls, 2022; Wakefield *et al*., 2010), there remains a need to scope the evidence on communications interventions; development of campaigns, their content, target audiences, theoretical underpinning, message framing with a precise focus on population-level breastfeeding communications campaigns delivered via mass media/social media specifically, and not individually mediated communications (such as group antenatal classes, peer support, individual lactation support). 

Graziose *et al.*’s (2018) systematic review of mass media interventions for infant and young child feeding (IYCF) in LMICs called for more consistent reporting of the detail of the design of interventions to better understand how, why, for whom and under what conditions mass media interventions for IYCF may be effective. From our preliminary searches of the databases, we have not identified any published guidance for intervention development on the common elements, components, specific content or message framing for mass media communications interventions on breastfeeding. This scoping review will inform the development of such guidance.

The goal of this scoping review is to describe the state of the evidence on mass media campaigns related to breastfeeding. This represents the first step towards generating theory-based and evidence-informed guidance on how to develop, implement and evaluate mass media campaigns on breastfeeding. Our first objective is to systematically identify and map the breadth, type and characteristics of the research on developing, implementing and evaluating mass media campaigns designed to influence breastfeeding behaviour.

Scoping reviews can serve several purposes. They can be conducted to identify the types of available evidence in a given field in a systematic and transparent way, as per our first objective. They can also be used as a precursor to a systematic review ( Munn *et al*., 2018; Munn *et al*., 2022; Pollock *et al*., 2024). If we identify sufficient evidence in our scoping review, we will then conduct a systematic review to critically appraise and synthesise the research on the effectiveness and implementation of campaigns.

The proposed scoping review will be conducted in accordance with the JBI methodology for scoping reviews ( Peters *et al*., 2020) and this protocol will be made publicly available on OSF.io and via HRB open. ^1^

## Review question

Overarching question: What research has been conducted that can inform the development, implementation and evaluation of mass media campaigns, in highly developed countries, aimed at influencing breastfeeding rates?

Sub-question 1: What strategies, processes or approaches were used to *develop* mass media campaigns aimed at influencing breastfeeding?
Sub-question 2: What, if any, theories, models or frameworks were reported to underpin the *development* of mass media campaigns aimed at influencing breastfeeding?
Sub-question 3: What were the *components* of mass media campaigns in terms of their source, message, channel, receiver and destination?
Sub-question 4: What theories, models or frameworks were reported to underpin the *implementation* of mass media campaigns aimed at influencing breastfeeding?
Sub-question 5: What strategies, processes or approaches were used to *implement* mass media campaigns aimed at influencing breastfeeding?
Sub-question 6: What research designs, methods and outcomes were used to evaluate the design, implementation and effectiveness of mass media campaigns aimed at influencing breastfeeding?

## Eligibility criteria

### Participants

The populations of interest are the general public and/or the people and places (i.e. the social and physical environment) through which mass media campaigns seek to directly or indirectly influence breastfeeding outcomes. These include, for example, campaigns aimed at grandparents, workplaces, young people who are not yet parents, public spaces, services, places of work, and broader social systems and institutions, at local and distal levels.

### Concept

Eligible articles should contain the following core concepts of interest:

1) A description of, or guidance on,
2) the design, implementation and/or evaluation of a mass media campaign/other mass communication campaign (whether delivered as a standalone intervention or as part of a package of interventions) that actively and systematically use mass media or other means of communication, such as social media, with the intent of influencing the health behaviour of a large target audience
3) with the aim of influencing breastfeeding at scale.

Articles missing any of the core concepts above will be excluded.

Note: We do not intend to include mHealth technologies as an eligible form of mass media for this review. We define mHealth as the use of mobile devices to support medical practice. We exclude mHealth technologies because scoping searches indicated that they tend to be used to improve access to and uptake of medical supports and require active consent of the message recipient to receive messages, whereas mass media campaigns require no such consent.

### Context

Eligible studies must be conducted in one or more of the 68 countries classified as ‘very high’ on the Human Development Index (HDI) in 2022, which is the most recent year with data available at the time of publication ( United Nations Development Programme, 2025; World Population Review, 2024). 

### Types of sources

Peer-reviewed and grey literature (including research theses) are both eligible for inclusion as long as they also contain at least one of the following features:

- Empirical research, which we take to refer to research whose data comes from real-world observations and experiments. This includes both primary research and secondary research such as evidence syntheses and narrative literature reviews.
- Non-empirical reports or documents that are intended as discussion papers, position papers, opinion pieces or which provide relevant models, frameworks or guidance.

We will exclude blogs, conference extracts, and news articles because they typically lack rigorous methodological frameworks and empirical data. Similarly, books and book chapters will be excluded unless they present original empirical research not published elsewhere.

### Language and publication date

Only studies published in English will be included.

Studies published since 1990 will be included as this was when the Innocenti Declaration On the Protection, Promotion and Support of Breastfeeding ( WHO/Unicef, 1990) was first produced, which was foundational for the Global Strategy on Breastfeeding ( WHO/Unicef, 2003).

### Knowledge user consultation

Knowledge users were engaged throughout this work and as part of the larger MaxSBF programme, including in the development of the research grant application. For this scoping review specifically knowledge users contributed through:

- structured conversations with two community-based knowledge users with expertise in breastfeeding support and the social and cultural influences on infant feeding decisions
- two expert methodologists in evidence synthesis
- pen and paper-based consultation with 30 lactation consultants who identified priority target groups for communications interventions and local factors that influence breastfeeding
- one local lead for child health development.

Knowledge user insights informed key decisions about the review, including the choice of methodology, shaping the inclusion and exclusion criteria, and key concepts included in data extraction. These consultations were essential in building a data extraction tool that could identify relevant information and gaps in the research evidence from the perspective of expert practitioners and knowledge users. In terms of JBIs guidance this involvement would meet the levels of controlling, influencing and contributing to the work. 

## Information sources

The information sources for this scoping review include:

- Databases and platforms of peer-reviewed literature
- Databases and websites of grey literature
- Citation chaining of included studies
- Directly contacting via email expert authors and organisations.

We will search for peer-reviewed literature and relevant evidence synthesis via PubMed, Embase, CINAHL, PsycInfo, Proquest, Academic Search Complete (EBSCO) and Cochrane Library. 

We will conduct forward and reverse citation searching of included studies and reviews and grey literature searches via web searches, OSF, and contact with known authors/ organisations in the field. The search will be undertaken in January/February 2025.

We will search for grey literature on Google, Google Scholar, Open Science Framework (OSF) and the websites of relevant organisations including the WHO, UNICEF, World Alliance for Breastfeeding Action, and the World Breastfeeding Trends Initiative.

We will conduct backward citation chaining by screening the reference lists of included studies and reviews and forward citation chaining by using ‘Citation Chaser’ ( Haddaway *et al.*, 2022) to screen other studies that reference the included articles.

A list of expert authors and organisations to be contacted will be developed as the review progresses, in consultation with knowledge users of the broader MaxSBF programme.

These information sources have been selected because of their relevance to the scoping review questions and their accessibility to the review team.

## Search strategy

Searches will be conducted of each information source by one review team member. Information sources with peer-reviewed literature will be searched first, followed by searching sources with grey literature, citation chaining and contacting expert authors.

### Peer-reviewed literature

There are two core concepts of interest to this review: (1) breastfeeding, and (2) mass communications and mass media. The search strategy for peer-reviewed literature will be conducted around these two core concepts over four stages.

In stage 1, two members of the review team (JH and KMG) conduct limited scoping searches on MEDLINE (PubMed) and Google Scholar to identify an initial bank of relevant articles on the topic (complete)

In stage 2, search terms are identified for the core concept of ‘breastfeeding’. We will use a verified search string developed using the ‘pearl harvesting’ methodology ( Sandieson, 2006) ^2^. Pearl harvesting is an exhaustive process that builds and tests a search string designed to maximise both sensitivity and specificity and remove redundant search terms ( Sandieson, 2006).

In stage 3, search terms for mass communications and mass media are gathered through identifying related terms used in the titles, abstracts and keywords and index terms of articles identified in stage 1.

In stage 4, the search terms identified in stages 2 and 3 are tested by an experienced evidence synthesis specialist (JH) in PubMed for sensitivity and specificity. The search strategy for PubMed is provided below, the concept is in bold, the search terms to capture that concept follow. This core strategy will be tailored to the notation and indexing practices of each database and the full list of searches will be provided in the scoping review.

**Breastfeeding:** ((infant N3 feeding) OR (neonatal N3 feeding) OR (breast N3 feeding) OR (breast N3 milk) OR (mother* N3 milk) OR (maternal N3 milk) OR (human N3 milk) OR (infant N3 lactation) OR (infant N3 nutrition*) OR (infant N3 food) OR (breastfeeding OR breastfed OR breastmilk))

AND

**Communication campaign**: digital media OR electronic media OR social media OR social network OR broadcast media or mass media or print media or radio or television or TV or newspaper OR news media OR online news OR digital technology or news media or online media OR health promotion campaign OR behaviour change communication OR communication campaign OR Social norm OR social marketing OR health marketing OR commercial milk formula marketing OR advert*

AND

**Evaluation, development or implementation:** efficacy OR effective* OR sample OR cross-section* OR design* OR develop* OR implement* OR intervention* OR "evaluation studies" OR "evaluation studies as topic" OR "program evaluation" OR "validation studies as topic" OR (pre- AND post-) OR (pretest AND posttest) OR (program* AND (evaluat* OR effectiveness)) OR intervention OR random* OR (controlled N3 trial*)

### Grey literature

The interfaces and search capabilities of grey literature databases are usually less sophisticated than those of peer-reviewed literature, requiring a simplified list of search terms to be used when searching. This simplified list will be based on the two core concepts of the review, breastfeeding and communication campaigns. However, this also risks increasing the sensitivity of the search to an impractical level for screening. To maintain feasibility, stopping criteria will be implemented to limit the screening of grey literature. Searches will be ordered by relevance, and at least 50 initial results will be screened before applying stepwise evaluation (every 25 results) to determine if screening should continue. The stopping criteria will be applied to searches on Google, Google Scholar, OSF.

Websites of WHO, UNICEF and will be searched by screening relevant webpages (e.g. ‘resources’ or ‘publications’) and using keyword searches if a search bar exists for the website.

## Source of evidence selection

Following the search, all identified citations will be collated and uploaded into EndNote x20 (Clarivate Analytics, PA, USA) and duplicates removed. Screening and data extraction will be supported by EPPI Reviewer Web Evidence Synthesis Software (Version: 6). Similar review management software, with more limited capabilities, is available free of charge via https://www.rayyan.ai/.

At title and abstract screening two reviewers will first independently screen at least 10% of the retained records to provide data to train EPPI reviewers’ priority screening tool. This tool uses text mining to list references in order of similarity to studies identified by human reviewers as ‘include’ on title and abstract. Once the tool is trained, we will switch to single screening. A further random sample of 10% of all excluded records will be screened by a second human reviewer for quality control. If automated screening performs poorly as the ‘second reviewer’ automated screening will be abandoned, and a second human reviewer will review each record.8u

Potentially relevant sources will be retrieved in full text, supported by EPPI reviewers Zotero link tool that can automatically identify and bulk import pdfs of full texts. The full text of selected citations will be screened against the inclusion criteria by two reviewers working independently. Reasons for exclusion of sources of evidence at full text that do not meet the inclusion criteria will be recorded and reported in the scoping review. Any disagreements that arise between the reviewers at each stage of the selection process will be resolved through discussion, or with an additional reviewer/s. The results of the search and the study inclusion process will be reported in full in the final scoping review and presented in a flow diagram, in line with guidance provided by the “Priority Items for Systematic Reviews and Meta-analysis Scoping review extension (PRISMA-ScR) checklist ( Tricco *et al*., 2018). Our completed PRISMA-ScR checklist is made available as extended data.

## Data charting process

*Data will be charted (extracted) from papers included in the scoping review by two or more reviewers working independently using a data extraction tool developed by the reviewers. As shown in Table 1, extracted data will include background information about the article itself (e.g. author(s), funder(s), type of evidence, etc.), as well as information about mass media campaigns for breastfeeding (e.g. the components of communications; development and implementation processes; underlying theories, models and frameworks, etc.). The items have been selected based on their relevance to the review questions.*

**Table 1.** Data charting items.

**Article Characteristics**	• Author(s) • Year published • Title • Type of Source • Purpose/aim of article • Funder/commissioner • Conflicts of interest declared
**Breastfeeding Mass Media ** **Campaign (MMC) Characteristics**	• MMC name, country, target audience setting, and type of MMC • Processes, approaches and strategies of MMC development and implementation • Theories, models or frameworks informing MMC development and implementation • Components of MMC (Target issue, outcomes and audience; Messaging core idea, message content, source, framing and channels of communication). • MMC evaluation design, methods and outcomes measured • Recommendations on the design, implementation or evaluation of MMCs

The data extraction tool we have developed will be piloted using five randomly selected included records and modifications agreed within the team. Any proposed modifications to the data extraction tool after this pilot period will be discussed and agreed within the review team. Any necessary modifications will be retrospectively applied to sources where data extraction was already completed. Modifications will be detailed in the scoping review. Data extraction will be conducted independently and in duplicate by two reviewers. Any disagreements that arise between the reviewers will be resolved through discussion, or with an additional reviewer/s. If appropriate and required, authors of papers will be contacted to request missing or additional data.

## Data analysis and presentation

Recent guidance published by the Joanna Briggs Institute ( Pollock *et al*., 2023) recommends several different methods for analysing and presenting the extracted data. These include the use of basic descriptive statistics, tabular presentation, graphical presentation and narrative summaries ( Pollock *et al*., 2023). To this, we also add a basic form of thematic analysis.

The evidence will be mapped and summarised in line with the objectives for the review and research question and sub questions. The precise format of presentation will be finalised after piloting and in consultation with knowledge users to maximise its usability. However, Table 2 shows the planned analysis and presentation methods at the time of developing this protocol. Any deviations from the protocol in the final scoping review will be reported with rationale.

**Table 2.** Data analysis and presentation methods for the scoping review.

Extracted Data Related to	Basic Descriptive Statistics	Tabular Presentation	Narrative Summary	Basic Thematic Analysis
Article Characteristics	✓	✓	✓	
Sub-Question 1	✓	●	✓	
Sub-Question 2	✓	●	✓	
Sub-Question 3	✓	●	✓	
Sub-Question 4	✓	●	✓	
Sub-Question 5	✓	●	✓	✓
Sub-Question 6	✓	●	✓	

✓ The data will be analysed and presented with this method in the main scoping review article. ● The data will be analysed with this method but the results will be presented in supplementary material rather than in the main scoping review article.

Data extracted on the characteristics of included articles and on all seven sub-questions of the review will be analysed and presented using basic descriptive statistics. Namely, the number of times specific strategies, processes, approaches, theories, models, frameworks, mass media campaign components, research designs, methods, and outcomes are reported in included articles will be presented as counts and percentages to demonstrate how frequently these items appeared in the included literature. We will describe the results of the basic descriptive statistical analysis with data exported from EPPI reviewer, with narrative summaries to help readers interpret the findings and their potential meanings.

Tables presenting information for all data extraction items will be developed to assist the review team to compare data across the included articles. However, we anticipate only a table on article characteristics will be provided in the main scoping review article, with the remainder provided as supplementary material. Finally, for mass media campaign content, if there is sufficient data available, we will conduct a basic thematic analysis of the content and framing of the messages of the mass media campaigns to understand the basic themes of messages that have been used to promote or undermine breastfeeding. One reviewer, with expertise in breastfeeding communication (JH) will assess how breastfeeding is presented in each campaign using both descriptions extracted from study reports and any available imagery from the campaign using the following steps:

1. Familiarisation with the relevant extracted data.
2. Inductively code extracted data and available campaign imagery, by recording initial thoughts on each extracted piece of data and assigning a short label that represents the reviewers interpretation of the content, message strategy and its framing.
3. Review the coded data to identify recurring patterns and themes across the codes.
4. Review, define and name the themes.
5. A second reviewer will then review the coded data and imagery.
6. Any disagreements or expansions on the coding will be discussed until consensus is reached or a third reviewer will provide the final decision.
7. Write basic analysis to summarise the prevalence of identified themes in included campaigns.

## Ethics and consent

Ethical approval and consent were not required.

## Data Availability

No data are associated with this article Extended data is available via OSF and includes the data extraction template, PRISMA-ScR Checklist and sample search strategy DOI 10.17605/OSF.IO/DXNSB { Hanratty, 2025 #7061}. Data are available under the terms of the Creative Commons Attribution 4.0 International license (CC-BY 4.0).
