# Supplementary material for: Population-level communication interventions for breastfeeding: Protocol for a scoping review
Source: HRB Open Res. 2025 Nov 17;8:76. Originally published 2025 Jul 7. [Version 2] doi: 10.12688/hrbopenres.14088.2 (PMC12504943; doi:10.12688/hrbopenres.14088.2)
Supplement: Peer review history

# Reviewer report: Reviewer response for version 2

**DOI:** 10.21956/hrbopenres.15725.r51527

**Published:** 2025-11-22

**Authors:** Henriette Knold Rossau

**Affiliations:** 1 University of Copenhagen, Copenhagen, Denmark

I am satisfied with the responses provided by the authors and look forward to reading the finalized scoping review. All the best

Is the study design appropriate for the research question?

Yes

Is the rationale for, and objectives of, the study clearly described?

Yes

Are sufficient details of the methods provided to allow replication by others?

Yes

Are the datasets clearly presented in a useable and accessible format?

Not applicable

Reviewer Expertise:

Complex interventions, public health, breastfeeding intervention, social inequalities in health

I confirm that I have read this submission and believe that I have an appropriate level of expertise to confirm that it is of an acceptable scientific standard.

# Reviewer report: Reviewer response for version 1

**DOI:** 10.21956/hrbopenres.15475.r48341

**Published:** 2025-10-09

**Authors:** Monique M. Turner

**Affiliations:** 1 Michigan State University, East Lansing, Michigan, USA

I think this scoping review is important and I am pleased to see this work being conducted.  But I do recommend that the authors extract additional data, when available, from the literature. 

 In communication, the essential ingredients to a successful campaign/intervention are: source, message, channel, receiver, objectives. 

 This scoping review will pull some of these ingredients--but for this review to be really good it needs to extract all of it.  WHO is the source of the message? WHAT is the message strategy (narrative, emotion (what emotion), facts, framing etc), who is the intended audience and what channel(s) are used.  I think these details will make this review much better.

Is the study design appropriate for the research question?

Partly

Is the rationale for, and objectives of, the study clearly described?

Yes

Are sufficient details of the methods provided to allow replication by others?

Yes

Are the datasets clearly presented in a useable and accessible format?

Not applicable

Reviewer Expertise:

communication, campaign design, message strategy, public health

I confirm that I have read this submission and believe that I have an appropriate level of expertise to confirm that it is of an acceptable scientific standard.

# Response

**Published:** 2025-11-05

**Authors:** Jennifer Hanratty

**Affiliations:** Centre for Effective Services, Dublin, Leinster, Ireland

Thank you for your review and expert constructive suggestions.

 We have included source, message, channel, receiver, and objectives in our data extraction tool. We have also included consideration of the intended audience and the subject(s) featured in the campaign. We welcome your suggestion of attempting to capture more detail on who the source is, the message strategy, emotion and framing. We will attempt to achieve this in the thematic analysis of campaign content. For reference, this section of the protocol now reads:

 "Finally, for mass media campaign content, if there is sufficient data available, we will conduct a basic thematic analysis of the content and framing of the messages of the mass media campaigns to understand the basic themes of messages that have been used to promote or undermine breastfeeding. One reviewer, with expertise in breastfeeding communication (JH) will assess how breastfeeding is presented in each campaign using both descriptions extracted from study reports and any available imagery from the campaign using the following steps:

1. Familiarisation with the relevant extracted data.
2. Inductively code extracted data and available campaign imagery, by recording initial thoughts on each extracted piece of data and assigning a short label that represents the reviewer's interpretation of the content, message strategy and framing.
3. Review the coded data to identify recurring patterns and themes across the codes.
4. Review, define and name the themes.
5. A second reviewer will then review the coded data and imagery.
6. Any disagreements or expansions on the coding will be discussed until consensus is reached or a third reviewer will provide the final decision.
7. Write a basic analysis to summarise the prevalence of identified themes in included campaigns."

Thank you again for sharing your expert advice.

# Reviewer report: Reviewer response for version 1

**DOI:** 10.21956/hrbopenres.15475.r50078

**Published:** 2025-10-07

**Authors:** Jessica L Schue

**Affiliations:** 1 Johns Hopkins Bloomberg School of Public Health, Baltimore, MD, USA

The article presents the protocol for a scoping review of mass media interventions used to promote breastfeeding in high income countries. The authors demonstrate a clear rational and need for the project, explaining that this project is a critical step in a multi-step process to develop normative guidance for high income countries. The research questions are broad and cover several aspects of a mass media campaign, from the development, methods, implementation, and research methods to evaluate campaigns.

 The article is well written and clear, there are no major concerns to be addressed. Minor comments include the following.

 Only articles and literature in English will be included, which may bias the findings towards cultures with predominantly english speakers. While historically the exclusion of articles based on language was reasonable given limited accessibility, recent computing advances have made this less necessary. If including non-english articles is not feasible during this scoping review, it should be considered for any subsequent systematic review.

 The second comment is related to quality control of the screening tool used. Do the authors have a specific threshold that must be met to accept the automated screening results?

 Overall, the article is clear and the methods are sufficiently described for reproducibility.

Is the study design appropriate for the research question?

Yes

Is the rationale for, and objectives of, the study clearly described?

Yes

Are sufficient details of the methods provided to allow replication by others?

Yes

Are the datasets clearly presented in a useable and accessible format?

Not applicable

Reviewer Expertise:

Maternal and child health, research design, demand generation.

I confirm that I have read this submission and believe that I have an appropriate level of expertise to confirm that it is of an acceptable scientific standard.

# Response

**Published:** 2025-11-05

**Authors:** Jennifer Hanratty

**Affiliations:** Centre for Effective Services, Dublin, Leinster, Ireland

Thank you for your through review and constructive suggestions.

 Point 1: We agree that including only English language publications has the potential to bias the review and will note that as a limitation in the scoping review and, as you suggest, consider expanding our inclusion criteria if a systematic review follows.

 Point 2: We did not set a specific threshold for automated screening, we will include this point in the discussion in the scoping review.

# Reviewer report: Reviewer response for version 1

**DOI:** 10.21956/hrbopenres.15475.r50077

**Published:** 2025-10-07

**Authors:** Louisiana Montserrat Sanchez

**Affiliations:** 1 University of Southern California, Los Angeles, CA, USA

**Summary of the article:**

 Thank you so much for the opportunity of reviewing this manuscript. The manuscript describes a protocol for a scoping review that will map and synthesize the evidence on population-level communication interventions for breastfeeding in very high Human Development Index (HDI) countries. The rationale is grounded in persistently low breastfeeding rates in high-income settings, the influence of social and cultural norms, and the absence of clear evidence-based guidance on designing effective mass communication interventions.

 The authors plan to search multiple databases (PubMed, Embase, PsycInfo, Proquest, etc.) and grey literature sources (Google, OSF, UNICEF, WHO), conduct citation chaining, and engage with knowledge users. Screening and extraction will be managed using EPPI Reviewer with automation features, and the PRISMA-ScR checklist will be followed. Extracted data will include interventions characteristics, theoretical underpinnings, message framing, conflicts of interest, and evaluation outcomes. Data will be summarized descriptively, tabulated, and analyzed thematically to identify recurring patterns in intervention design and messaging.

 The protocol is robust and methodologically aligned with best practices, but a few refinements could improve reproducibility and clarity.

 Responses to key questions:

 1. Is the rationale for, and objectives of, the study clearly described? Yes, the introduction provides strong justification, citing epidemiological evidence, socio-ecological frameworks, and gaps in existing reviews. The objectives and six sub-questions are clearly presented, offering a roadmap for the scoping review.

 2. Is the study design appropriate for the research question? Yes, a scoping review is the correct approach for mapping breadth and characteristics of the literature. The design is consistent with JBI and PRISMA-ScR guidance, and the authors appropriately note that a systematic review may follow if sufficient evidence is identified.

 3. Are sufficient details of the methods provided to allow replication by others? Partly, the manuscript provides substantial methodological detail (1) search and selection, databases and grey literature sources and listed; a sample PubMed strategy is included; PRISMA-ScR compliance is noted. (2) screening and extraction, independent and duplicate review is planned; EPPI Reviewer will be used; piloting and consensus procedures are described. (3) data analysis, descriptive statistics, narrative summaries, and thematic analysis are planned, guided by JBI recommendations.

 Gaps that limit full reproducibility, include:

1. definitions of interventions. Box 1 offers useful definitions, but operational boundaries between “mass communication,” “mass media,” and “social media” are blurred. The authors should add explicit examples of eligible versus ineligible interventions (e.g., a systematic social media campaign vs. a static informational website; a broad Facebook push vs. mHealth opt-in texts).
2. Standardized procedures. Thematic analysis steps are described, but details on coder reliability, consensus procedures, and sharing of coding frameworks are lacking. Clarifying these would ensure reproducibility.
3. Data management. While EPPI Reviewer is identified, the manuscript does not specify whether descriptive statistics will be generated within EPPI or exported, nor whether all variables will be tabulated. Greater detail would strengthen transparency.
4. Conflict of interests coding. The authors note conflict of interest  will be captured, but not detail is provided on how funders/sponsors will be categorized (governments, NGO, commercial, academic). Given the political economy of formula marketing, this is an important addition.

I recommend that authors: (1) specify whether they will extract declared and undeclared conflicts of interests, (2) clearly categorize the type of sponsor/funder (e.g., government, NGO, academic, commercial/industry), consider noting whether industry-affiliated authorship or partnerships are reported, as these can influence the framing of communication campaigns.

 Adding this structure will improve transparency and allow the review to capture critical contextual differences between government/NGO-led breastfeeding promotion campaigns versus commercially influenced campaigns.

 4. Are the datasets clearly presented in a usable and accessible format? Not application, this is a protocol, no results are yet available. However, the authors provided extended data on OSF, including the data extraction template, PRISMA-ScR checklist, and sample search strategy, which is appropriate and accessible.  

Major points to be addressed: (1) clarify definitions in Box 1, provide concrete examples of what counts as an eligible intervention versus excluded forms (e.g., broad social media campaigns vs. passive informational websites); (2) detail thematic analysis procedures, specify how coding will be conducted, inter-rater reliability assessed, and disagreements resolved. Consider committing to share the coding framework as supplementary data; (3) expand on data management plans, clarify whether descriptive analysis will be performed in EPPI reviewer or another platform, and which variables will be reported in the main article versus supplementary material; (4) enhance conflict of interest/funding coding, explicitly state how conflicts of interests and funding sources will be categorized, for example, government, NGO, academic, commercial.

Minor points: (1) readability, some section of the introduction could be streamlined for clarity; (2) Box 1 presentation, presenting the definitions in a tabular form with columns for term/definition/example could make them more easy to read; (3) knowledge user engagement, the manuscript described knowledge user consultation, but linking this directly to data extraction items (e.g., why particular variables were prioritized) would strengthen justification.

 This is a timely and well-prepared protocol that addresses a significant public health gap. With minor but important clarification, particularly in definitions, coding, and data management, the protocol will be fully reproducible and scientifically sound.

Is the study design appropriate for the research question?

Yes

Is the rationale for, and objectives of, the study clearly described?

Yes

Are sufficient details of the methods provided to allow replication by others?

Partly

Are the datasets clearly presented in a useable and accessible format?

Not applicable

Reviewer Expertise:

Maternal and child health; breastfeeding promotion; population-level and social media communication interventions; evidence synthesis (scoping and systematic reviews); health behavior change

I confirm that I have read this submission and believe that I have an appropriate level of expertise to confirm that it is of an acceptable scientific standard, however I have significant reservations, as outlined above.

# Response

**Published:** 2025-11-05

**Authors:** Jennifer Hanratty

**Affiliations:** Centre for Effective Services, Dublin, Leinster, Ireland

Thank you for your expert review and the obvious attention to detail. In response to your comments and suggestions:

 Point 1: We have revised box 1 to add specific examples. Thank you for the suggestions.

 Point 2: Thank you, we have revised our approach and simplified our thematic analysis, removing reference to Braun and Clarke and adding detail on consensus procedures. The section now reads:

 "Finally, for mass media campaign content, if there is sufficient data available, we will conduct a basic thematic analysis of the content and framing of the messages of the mass media campaigns to understand the basic themes of messages that have been used to promote or undermine breastfeeding. One reviewer, with expertise in breastfeeding communication (JH) will assess how breastfeeding is presented in each campaign using both descriptions extracted from study reports and any available imagery from the campaign using the following steps:

1. Familiarisation with the relevant extracted data.
2. Inductively code extracted data and available campaign imagery, by recording initial thoughts on each extracted piece of data and assigning a short label that represents the reviewer's interpretation of the content, message strategy and framing.
3. Review the coded data to identify recurring patterns and themes across the codes.
4. Review, define and name the themes.
5. A second reviewer will then review the coded data and imagery.
6. Any disagreements or expansions on the coding will be discussed until consensus is reached or a third reviewer will provide the final decision.
7. Write basic analysis to summarise the prevalence of identified themes in included campaigns."

Point 3: We have added “calculated with data exported from EPPI reviewer”. The variables that will be reported in the main article vs supplementary material are specified in table 2.

 Point 4: Thank you, we agree on the importance of highlighting the source of funding in this context; however, we did not use pre-specified categories of funder. We planned to extract information verbatim from papers. In practice, we categorised funder types post-hoc so it would be disingenuous to add categories to the protocol.

 We added “declared conflicts of interest” to specify that we focused on those rather than undeclared. In practice we examine declared conflicts of interest in the context of the funding source to gain a more full picture of potential conflicts but we didn’t go into the level of detail of analysing the affiliation of individual authors due to resource limitations.

 Minor points: Thank you, we have reviewed and edited to improve readability throughout.

# Reviewer report: Reviewer response for version 1

**DOI:** 10.21956/hrbopenres.15475.r48637

**Published:** 2025-09-05

**Authors:** Henriette Knold Rossau

**Affiliations:** 1 University of Copenhagen, Copenhagen, Denmark

Thank you for inviting me to review the article: **Population-level communication interventions for breastfeeding: Protocol for a scoping review**

 The article describes the protocol for conducting a scoping review as a first step towards developing a guidance to inform development of population-level communications interventions for breastfeeding in high-income countries.

 The article is well written and meticulously describes the methodology for conducting the scoping review. As such, I have very few comments.

 The first comment pertains to the perspective of population-level communication interventions in whole. While such interventions may be beneficial for information dissemination, they risk strengthening health behaviours (such as breastfeeding) among socially advantaged groups and leave the more disadvantaged behind, thereby augmenting the social gap in health behaviours and -outcomes. The authors refer to the socioecological model in their article, which ensures me that this perspective will be included in subsequent research. I suggest supplementing the model with the so-called Rainbow Model (Dahlgren and Whitehead, Public Health 2021).

 The second comment pertains to the search strategy section. When describing the stages in the review process, present and past tenses are mixed. I would make for a better reading experience if tenses were aligned. Moreover, the three search strings for **Breastfeeding, Communication campaign** and **Evaluation, development or implementation **all stem from PubMed searches according to the description. However, all of them are different in their layout with the third appearing to be the most descriptive due to the subscripts (tiab, mesh etc). Could they be aligned, perhaps by providing the reader with the most ‘readable’ like the first string, and supplementing with the extensive strings online or elsewhere? And could a similar ‘string’ be included for the Grey literature section? Just to exemplify.

 The third comment is for the Source of evidence selection section. This is thoroughly described but could be supplemented by informing the reader what the proceeding will be if the quality control of the automated screening appears to be poor (“A further random sample of 10% of all excluded records will be screened by a second human reviewer for quality control.”).

 For nitty gritty details, there appears to be a full stop misplaced in this sentence: “Screening and data extraction will be supported by EPPI Reviewer Web Evidence Synthesis Software (Version: 6)., similar review management software, with more limited capabilities, is available free of charge via https://www.rayyan.ai/.”

 But overall, I wish the authors best of luck in their endeavours. The manuscript is of high quality and nearly ready for publication. I do not have to see it again before finalisation but will look forward to reading the scoping review when it is completed and published.

Is the study design appropriate for the research question?

Yes

Is the rationale for, and objectives of, the study clearly described?

Yes

Are sufficient details of the methods provided to allow replication by others?

Yes

Are the datasets clearly presented in a useable and accessible format?

Not applicable

Reviewer Expertise:

Complex interventions, public health, breastfeeding intervention, social inequalities in health

I confirm that I have read this submission and believe that I have an appropriate level of expertise to confirm that it is of an acceptable scientific standard.

## References

[1] : The Dahlgren-Whitehead model of health determinants: 30 years on and still chasing rainbows. *Public Health* .2021;199: 10.1016/j.puhe.2021.08.009 20-24 10.1016/j.puhe.2021.08.009 34534885

# Response

**Published:** 2025-11-05

**Authors:** Jennifer Hanratty

**Affiliations:** Centre for Effective Services, Dublin, Leinster, Ireland

Thank you for your detailed comments and constructive suggestions.

1. Your point on the potential for population-level communication interventions to widen inequalities in health is pertinent and thank you for suggesting Dahlgren G, Whitehead M. The Dahlgren-Whitehead model of health determinants: 30 years on and still chasing rainbows. Public Health. 2021 Oct;199:20-24. doi: 10.1016/j.puhe.2021.08.009. Epub 2021 Sep 14. PMID: 34534885. We will incorporate this model into the scoping review itself rather than tokenistic inclusion in the protocol at this point.
2. We have reviewed tenses and kept to present and future tense throughout the description of the search strategy and streamlined the presentation of the search strings in the main article text to make them more readable, thank you for this suggestion.
3. We have added that “if automated screening performs poorly as the ‘second reviewer’ automated screening will be abandoned, and a second human reviewer will review each record”
4. Thank you for spotting this, the errant full stop has been corrected.
